# Improved Edge Pixel Resolution in Modular PET Detectors with Partly Segmented Light Guides

**DOI:** 10.3390/s25227062

**Published:** 2025-11-19

**Authors:** Henry Maa-Hacquoil, Harutyun Poladyan, Brandon Baldassi, Borys Komarov, Janos Rado, Oleksandr Bubon, Alla Reznik

**Affiliations:** 1 Department of Physics, Lakehead University, Thunder Bay, ON P7B 5E1, Canada; hpoladya@lakeheadu.ca (H.P.); jrado@uwaterloo.ca (J.R.); sasha.bubon@radialismedical.com (O.B.); 2 Radialis Inc., Thunder Bay, ON P7A 7T1, Canada; brandon.baldassi@radialis.com (B.B.); borys.komarov@radialis.com (B.K.); 3 Thunder Bay Regional Health Research Institute, Thunder Bay, ON P7B 7A5, Canada; 4 Ontario Institute for Cancer Research, Toronto, ON M5G 0A3, Canada

**Keywords:** organ-targeted PET, detectors, light guide, partly segmented light guide

## Abstract

Background: The asymmetric distribution of optical photons near the edges of Positron Emission Tomography (PET) sensor modules introduces errors in the coordinate reconstruction of scintillation events when center-of-gravity (CoG) algorithms are utilized. This issue, sometimes referred to as the “edge effect”, results in overlap of crystal pixel signatures in flood maps and potential image artifacts in reconstructed PET images. Methods: Partly segmented 5 mm thick borosilicate light guides with slits cut parallel to the edges are filled with barium sulfate to restrict the spread of optical photons near the edges of the light guide. Data acquisitions are performed using single PET sensor modules in coincidence, both with single and multiplexed channel readout. CoG and truncated center-of-gravity (TCoG) methods are used for coordinate reconstruction. Results: A 22 × 22 array of crystal signatures are distinguishable on crystal flood maps produced using sensor modules with solid light guides and 24 × 24 arrays can be identified when using a partly segmented light guide. The pixel resolution around the edges and corners of the flood map is further improved when TCoG is used for coordinate reconstruction. Summary: We show that the introduction of a partly segmented light guide greatly improves coordinate reconstruction accuracy at the edges of a sensor module. In addition, it is demonstrated that the partly segmented light guides can be used in parallel with other proposed methods designed to fix the “edge effect”, including TCoG, to further coordinate reconstruction improve accuracy and crystal flood map quality.

## 1. Introduction

Silicon Photomultipliers (SiPMs) became a transformative technology in the field of Positron Emission Tomography (PET) imaging due to their ultra-high gain capability, which previously was the exclusive domain of bulky, low quantum efficiency vacuum photomultipliers (PMTs) [1,2,3]. The replacement of PMTs with SiPMs in PET technology enabled groundbreaking innovations such as time-of-flight (TOF) PET, PET/MRI (Magnetic Resonance Imaging), total-body PET and high-performing organ-targeted PET scanners. The clinical adoption of these technologies enables reduced radiation dose, improved image quality and enhanced diagnostic capabilities [1,4,5,6].

SiPMs are solid-state photodetectors composed of an array of Geiger-mode microcells, interconnected in parallel through individual current-limiting (or quenching) resistors to a common electrode. In analog SiPMs (commonly used in PET), the output signal is an analog sum of all individual microcell signals. Although each microcell operates in Geiger mode, the SiPM operates in proportional mode, where the amplitude of the output signal in individual SiPM channel is linearly proportional to the total number of microcells fired, reflecting the number of incident light photons [7].

Use of SiPMs allows us to preserve a commonly used modular (or block) detector design in PET technology where closely packed, small crystal elements are optically coupled to a matching array of photosensors. There are two basic configurations to couple discrete scintillation crystals to photodetectors, namely one-to-one coupling and light sharing.

In the one-to-one coupling configuration, each scintillation crystal element is directly coupled to an individual SiPM pixel. By carefully choosing the crystal and SiPM pixel sizes, the spatial resolution can be tuned to meet the requirements of high-resolution imaging applications, such as brain or small-animal PET systems. However, this design requires a higher number of readout channels and associated electronics, increasing overall system complexity and cost. The cost and complexity associated with one-to-one SiPM pixel readout can be mitigated by combining signals from multiple SiPMs into fewer readout channels using multiplexing techniques—a frequently used approach, especially when the scintillation pixel size is smaller than that of the individual photodetectors [8,9,10,11,12,13,14].

In the light-sharing configuration, optical photons generated in one crystal element are collected in more than one photodetector. For this, a light guide (commonly made from glass or acrylic) interfaces the scintillation crystal and SiPMs array to distribute scintillation photons from single crystal across several SiPM pixels. A weighted distribution algorithm, commonly referred to as Anger logic [15], is then applied to estimate the interaction coordinates of the gamma photon.

Typically, a center-of-gravity (CoG) algorithm, which is simply a generalization of Anger logic, is used for coordinate reconstruction. The general formula for CoG is described in Equation (1), where Xc and Yc are the scintillation event coordinates, and mi and mj are the signal amplitudes [16]:

(1) $$X_{c}\;=\;\frac{{\sum_{i}}^{n}m_{i}\;\times \;x_{i}}{{\sum_{i}}^{n}m_{i}},\;Y_{c}=\;\frac{{\sum_{i}}^{n}m_{i}\;\times \;y_{i}}{{\sum_{i}}^{n}m_{i}}$$

By implementing Anger logic and multiplexing strategies, PET detectors significantly reduce the number of required readout channels, thereby lowering system costs—an essential consideration for large area PET detectors [17,18]. However, light-sharing can introduce challenges. The CoG algorithm relies on the assumption that optical photon distribution is symmetric around the gamma photon interaction point [19]. Modular detector designs typically use a reflective coating, such as enhanced specular reflectance (ESR), that reflect optical photons off the detector edges. This approach becomes problematic for the CoG algorithm, as the use of ESR destroys this assumption near the detector module edges, where a significant amount of scintillation light is redirected toward the module’s center [19,20]. Consequently, the resulting asymmetric light spread biases the estimated interaction coordinates, reducing event localization accuracy around the module periphery [21,22].

Electronic noise further contributes to positional inaccuracies by distorting pixel signatures in crystal flood maps and diminishes the effectiveness of center-of-gravity-based reconstruction algorithms [23]. Consequently, modular SiPM-based PET detectors suffer from reduced spatial resolution at the edges and frequent misassignment of events to scintillation crystal pixels. This inaccurate edge pixel assignment is often referred to as the “edge effect” in PET devices [17,21]. The edge effect results in a reduction in crystal identification and degradation of spatial resolution [21].

Several strategies have been explored to improve coordinate reconstruction accuracy near the detector edges. One of the most direct solutions involves implementing one-to-one coupling between the scintillation crystal and SiPM arrays. Studies indicate that single channel readout, combined with noise suppression techniques, significantly enhances edge pixel resolvability [23]. However, this approach increases detector cost by necessitating a greater number of readout channels.

Modifications to the CoG coordinate reconstruction algorithm have also been proposed as a means of enhancing edge pixel resolution. This includes truncated center-of-gravity (TCoG) and position-weighted center-of-gravity (PW-CoG) methods that employ adjusted weighting functions to refine coordinate estimation for edge pixels [18,24,25]. Both algorithms have demonstrated moderate improvements in reconstruction accuracy but do not fully resolve the edge localization issue, with edge and corner pixel signatures still appearing merged [17,24,25].

An alternative approach to improve the resolvability of edge pixels involves modifying the light guide to manipulate the distribution of optical photons at the edges of the sensor module. Multiple studies have shown that modifying the light guide by changing the shape of the guide or introducing internal optical barriers improves edge pixel resolution [26,27,28,29,30]. One method to achieve this is by introducing reflective material within the light guide, positioned perpendicular to and in direct contact with the bottom face of the scintillator. These barriers restrict the lateral spread of photons toward the detector’s center, effectively funneling them downward. A small distance is left between the slit and photo detector to allow for some light sharing to still occur. This method has the advantage of being a passive solution, meaning that it does not require active electronic modifications, thereby maintaining system simplicity and reliability.

This study aims to demonstrate that a thick (5 mm), partly segmented light guide (PSLG) can enhance edge pixel coordinate reconstruction accuracy without requiring major electronic hardware modifications. By optimizing slit placement within the light guide in a modular SiPM-based PET detector, we aim to restrict the lateral spread of photons toward the module’s center, thereby enhancing edge pixel resolvability and improving overall spatial resolution. Additionally, we show that incorporation of an optimized segmented light guide can be used in conjunction with TCoG methods to take advantage of both methods to improve coordinate reconstruction accuracy. The effectiveness of combining both methods is assessed by measuring the crystal resolvability in the reconstructed crystal maps using Radialis Low-Dose (LD) organ-targeted PET technology [6,31].

## 2. Materials and Methods

### 2.1. Sensor Modules

**Figure 1:**
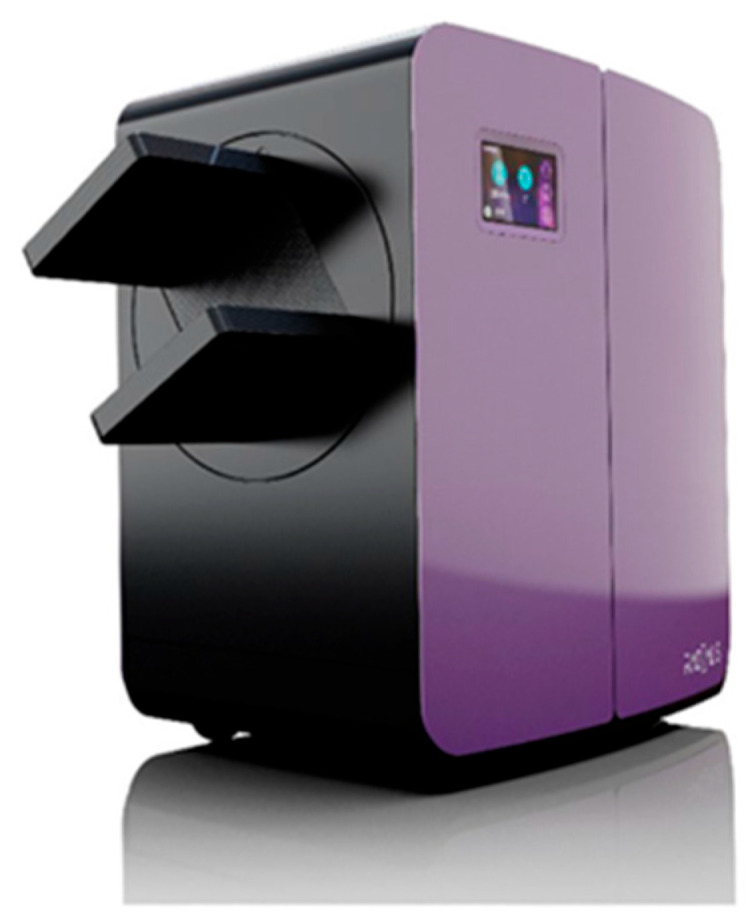
Radialis Low-Dose PET system with planar detector heads [31].

The Radialis Low-Dose PET (LD-PET) system (Figure 1) is an organ-targeted PET designed to detect small lesions using short acquisition times, while utilizing radiotracer doses up to ten times lower than those used in conventional whole-body PET systems, thereby enhancing patient safety and comfort.

As shown in Figure 1, the Radialis PET system is a compact scanner on wheels, with two large area (174 × 232 mm^2^), high-resolution planar detector heads positioned on opposing sides of the target organ to achieve comprehensive coverage during image acquisition. Placing detector heads close to an organ subtends large solid angle for efficient gamma ray detection and reduces signals from elsewhere in the body, thus increasing the signal-to-noise ratio and improving image contrast.

**Figure 2:**
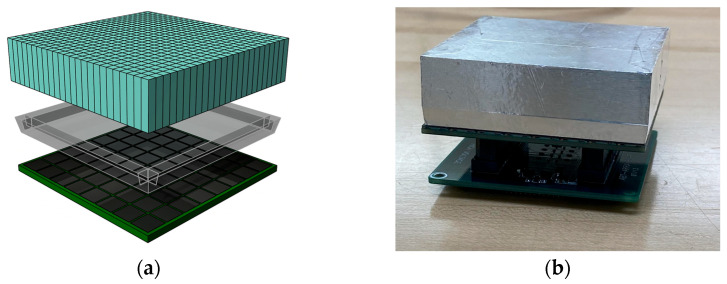
(**a**) Schematic drawing; (**b**) photo of LD-PET sensor module with electronic board underneath.

**Figure 3:**
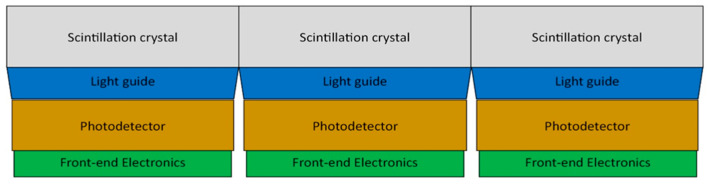
Diagram of tileable structure of sensor modules in detector head [6].

Each detector head comprises a 3 × 4 array of four-sided tileable sensor modules (Figure 2 and Figure 3) that are seamlessly tiled against each other to form a planar sensing area. Sensor modules contain a cerium-doped lutetium yttrium orthosilicate (LYSO) scintillation crystal array optically coupled to an array of SiPMs via a borosilicate light guide. The LYSO scintillation crystal array is composed of 24 × 24 square pixels, each measuring 2.32 mm × 2.32 mm × 13.0 mm with a 2.4 mm × 2.4 mm pitch, for a total crystal size of 57.6 mm × 57.6 mm. All of the outermost surfaces of the LYSO array—except the one facing the light guide—are covered with a specular reflector to maximize light collection efficiency.

The SiPM photodetector array consists of an 8 × 8 grid of ON Semiconductor (Phoenix, AZ, USA) Array Type-C pixels, with pixel pitch of 7.2 mm × 7.2 mm forming an optical sensing area of 56.6 mm × 56.6 mm. The scintillator and photodetector arrays are optically coupled using a tapered borosilicate light guide, with the top face precisely matching the scintillator dimensions (57.66 mm × 57.66 mm) and the bottom face aligned to the SiPM array size (56.40 mm × 56.40 mm) [6,31].

### 2.2. Partly Segmented Light Guide (PSLG) Design

**Figure 4:**
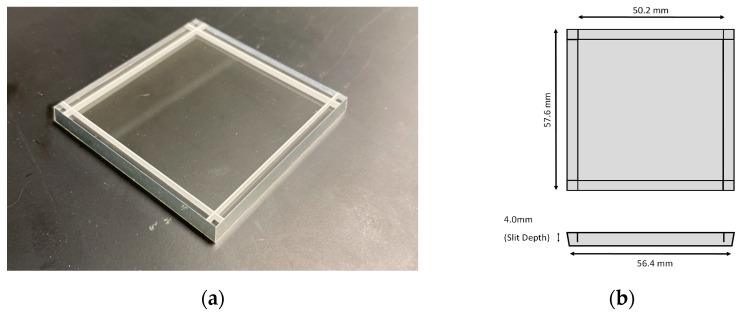
(**a**) Partly segmented borosilicate light guide with barium sulfate slits; (**b**) PSLG diagram with top and side view.

The borosilicate light guide is 5.0 mm thick; its edges are tapered (Figure 4) to match the dimensions of the LYSO and SiPM arrays. The lateral surfaces (sides) of the light guide are wrapped in ESR to minimize optical photon loss. To improve the resolvability of edge pixels, the light guide is partly segmented, as shown in Figure 4. The segmentation involves four slits cut into the borosilicate light guide, parallel to each edge of the light guide. The slits are positioned directly above the center of the second LYSO pixel from the edge of the sensor module to isolate the edge pixels from those in the crystal interior. The slits are approximately 0.3 mm wide to minimize interference with optical photon spread from other LYSO pixels. The insides of the slits are filled with barium sulfate due to its high reflectivity and relative ease of application. To gauge the effect of slit depth, light guides are engineered with slit depths of 2.5 mm, 3.0 mm and 4.0 mm, corresponding to 50%, 60% and 80% of the light guide thickness. These slit depths were selected based on values previously reported to significantly improve edge pixel resolution in detectors with similar architecture but thinner light guides and LYSO-to-SiPM pixel ratios of approximately 2.75 to 1 and 3 to 1 [28,29]. Our LD-PET detector utilizes a thicker light guide and LYSO-to-SiPM pixel ratio of 3 to 1, and therefore, we used an equivalent or larger slit depth-to-light guide thickness ratio to enhance pixel separation. Since some light sharing must remain near the edges to allow accurate CoG and TCoG calculation, slits with depths beyond 80% of the light guide thickness were not considered.

### 2.3. CoG and TCoG Coordinate Reconstruction Methods

**Figure 5:**
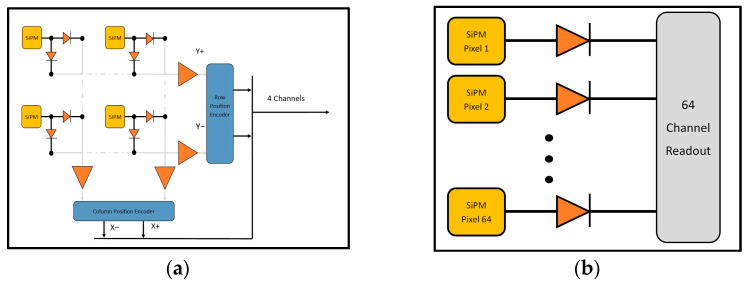
(**a**) Schematics of 64-to-4 multiplexed channel readout (**b**) schematics of single channel readout.

The CoG calculation for the LD-PET imager measures the sum of the signal across all the columns and rows of the SiPM array (Figure 5a). A weighted difference in the column or row is calculated and normalized to the total signal, retuning the center of the distribution in that direction. The x and y coordinates of the scintillation event are calculated using the following formulas, respectively:

(2) $$X_{pos}=\;\frac{X^{+}-X^{-}}{X^{+}-X^{-}},\;Y_{pos}=\;\frac{Y^{+}-Y^{-}}{Y^{+}-Y^{-}}$$

where X_pos_ and Y_pos_ are the x and y coordinates, and X^+^, X^−^, Y^+^, Y^−^ are the four preamplifier channel readouts.

The CoG calculations for the individual channel readout system are performed over 64 SiPM channels (Figure 5b). The formulas that account for the summation of over 64 channels for the x and y coordinates of the CoG for the light distribution are defined as follows:

(3) $$X\;=\;\frac{{\sum_{i=1}}^{64}\;m_{i}x_{i}}{{\sum_{i=1}}^{64}\;m_{i}},\;\;Y\;=\;\frac{{\sum_{i=1}}^{64}\;m_{i}y_{i}}{{\sum_{i=1}}^{64}\;m_{i}}$$

For a given SiPM pixel, x_i_ and y_i_ are the center coordinates of the corresponding pixel and m_i_ is the collected charge [17].

In the TCoG calculation, the collected charges are determined as follows. A preset fraction of the largest signals in the SiPM tile, defined as the truncation threshold f, is subtracted from all SiPM pixel signals. Channels with positive values are used for scintillation coordinate reconstruction, and are defined by the following formulas:

(4)                              X = ∑i=164 miTxi∑i=164 miT,       Y = ∑i=164 miTyi∑i=164 miT, miT = mi − f∗maxm1, m2, …m64,             ∣mi >f∗maxm1, m2, …m64                                                      0,             ∣mi ≤ f∗maxm1, m2, …m64 

T_mi_ is defined as the truncated weight of the analog signals from SiPM array [17].

The truncation threshold is empirically determined to balance noise suppression and sensitivity to low-energy events and is selected to be 0.0605. In such a way, TCoG mitigates noise near detection thresholds (common at edges due to photon loss) by truncating low-amplitude signals that disproportionately affect edge resolution.

### 2.4. Evaluation Methods

The crystal resolvability for each light guide design was assessed by acquiring and reconstructing crystal maps (flood histograms)—a two-dimensional histogram that displays the spatial distribution of detected scintillation events across the photodetector array. Each distinct peak in the crystal map corresponds to a single scintillation crystal in the array, and the clarity and separation of these peaks directly reflect the system’s ability to accurately localize gamma photon interactions within individual crystals.

For the crystal maps acquisition, a Na-22 point source with an activity of approximately 1.8μCi was placed at the center between two sensor modules, separated by 150 mm and aligned using a 3D-printed stand. To eliminate interference from ambient light, the modules were housed within a light-tight enclosure. All experiments were performed at room temperature. Data was collected in coincidence mode with each acquisition lasting 10 min. Only events within a 350–750 keV energy window were used for coordinate reconstruction.

Crystal maps are generated from each singles event corresponding to a valid coincidence event from the acquisition. Each crystal map contains 144 bins in both x and y directions, corresponding to a bin size of 0.4 mm × 0.4 mm. Three different coordinate reconstruction methods were used to create data for crystal mapping. The baseline crystal map is generated using a multiplexed 64-to-4 channel readout with the standard CoG algorithm, which represents the most common and cost-efficient approach. In addition, an acquisition is performed with single channel readout using CoG, and an acquisition with single channel readout and TCoG—the two more complex and costly readout methods utilized to maximize spatial accuracy and pixel resolvability for edge and corner regions.

## 3. Results

**Figure 6:**
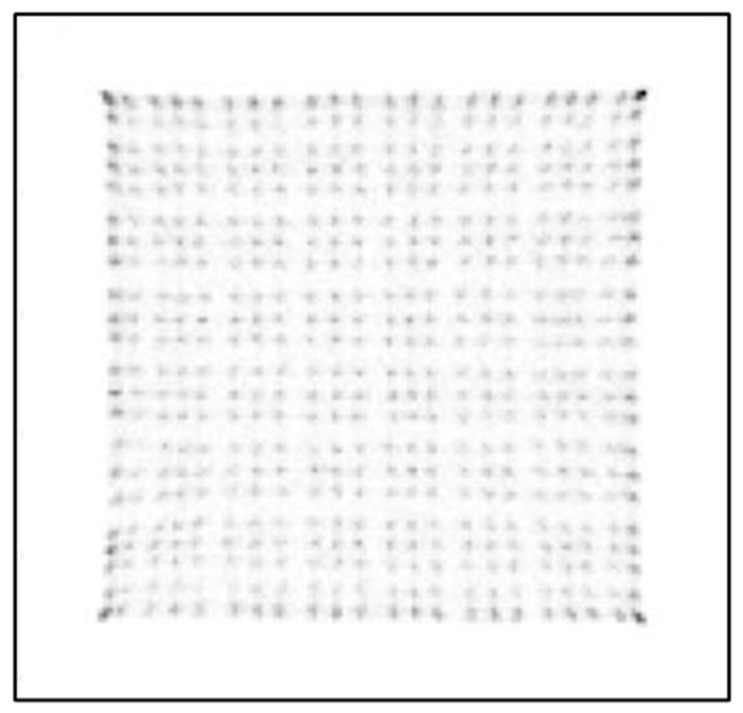
Crystal map generated from module with solid segmented light guide using multiplexed system and CoG.

Figure 6 presents a crystal map acquired using two sensor modules employing the traditional CoG method with a solid (non-segmented) borosilicate light guide. As can been seen from this figure, only 22 × 22 LYSO crystal elements are clearly distinguishable, with merged outermost rows and columns. The crystal map appears compressed in comparison with actual dimensions and the true 24 × 24 grid of the LYSO crystal array is not fully represented.

**Figure 7:**
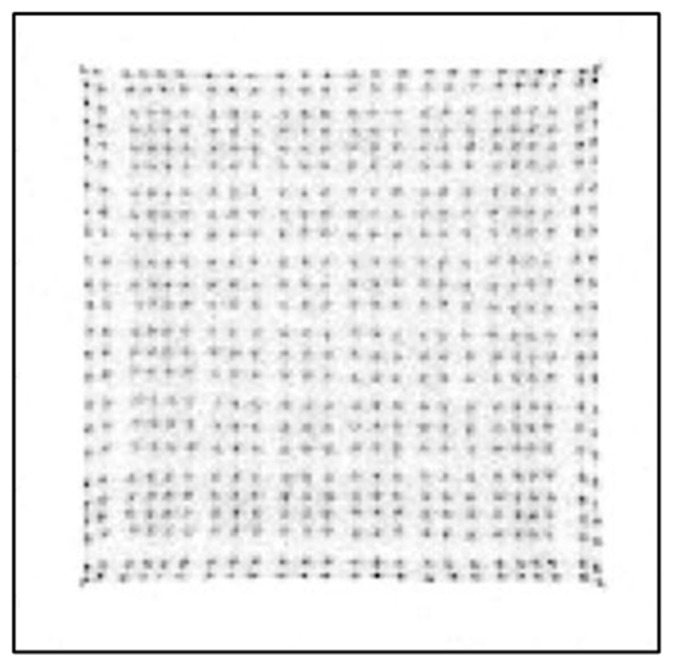
Crystal map generated from module with partly segmented light guide with 4.0 mm deep slits using multiplexed system and CoG.

**Figure 8:**
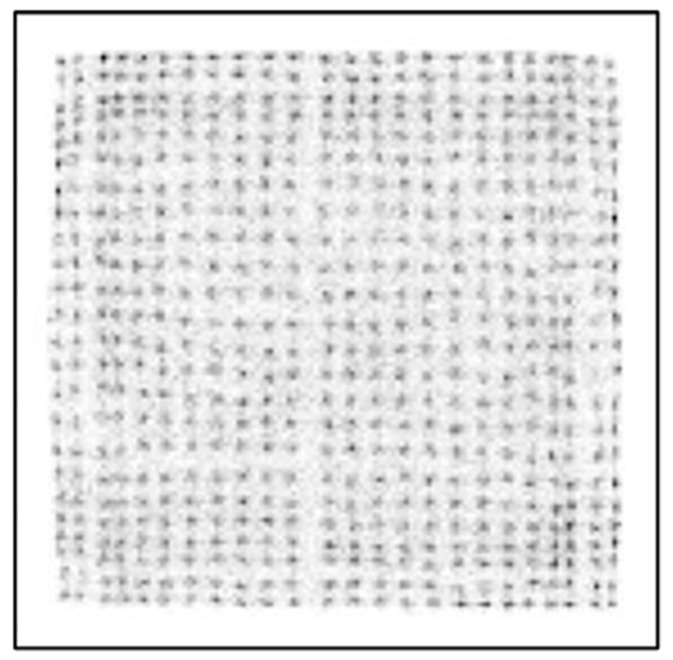
Crystal map generated from module with partly segmented light guide with 4.0 mm deep slits using single channel readout and CoG.

**Figure 9:**
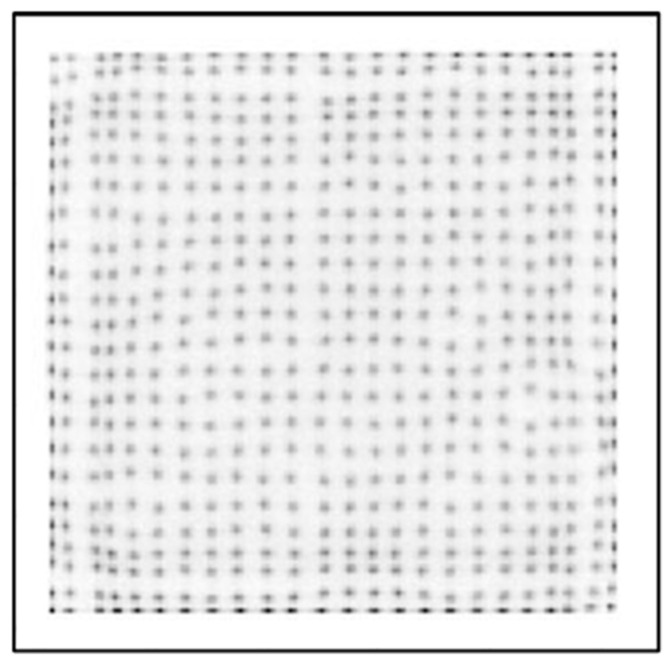
Crystal map generated from module with partly segmented light guide with 4.0 mm deep slits using single channel readout and TCoG. An “f” value of 0.0605 is used.

**Figure 10:**
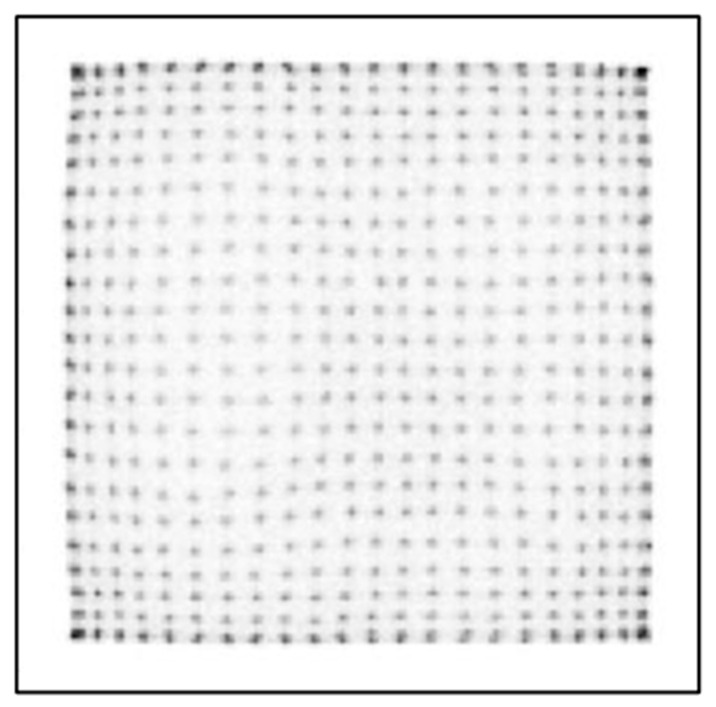
Crystal map generated from module with solid light guide using single channel readout and TCoG. An “f” value of 0.0605 is used.

Figure 7 and Figure 8 show crystal maps created using a PSLG with 4.0 mm slits again using the traditional CoG method on a multiplexed and single channel readout system, respectively. The significant improvement in resolvability of edge pixels achieved by incorporating the PSLG is apparent: both Figure 7 and Figure 8 reveal a full 24 × 24 grid of clearly resolved crystal signatures. With single channel readout (Figure 9), the spatial representation of the crystal map is much more uniform, with minimal compression at the periphery and overall. Figure 10 illustrates the effect of using the TCoG method in place of the traditional CoG with a solid light guide and single channel readout. While TCoG offers some improvement in homogeneity of a crystal map, only a 22 × 22 array of crystal elements is visible with edge pixels remaining superimposed. In contrast, Figure 9 demonstrates the multiplying effect of combining a PSLG with single channel readout and TCoG. This crystal map exhibits reduced noise compared to the single channel readout with standard CoG, and crucially, it resolves all pixels in each column and row (Figure 9).

**Figure 11:**
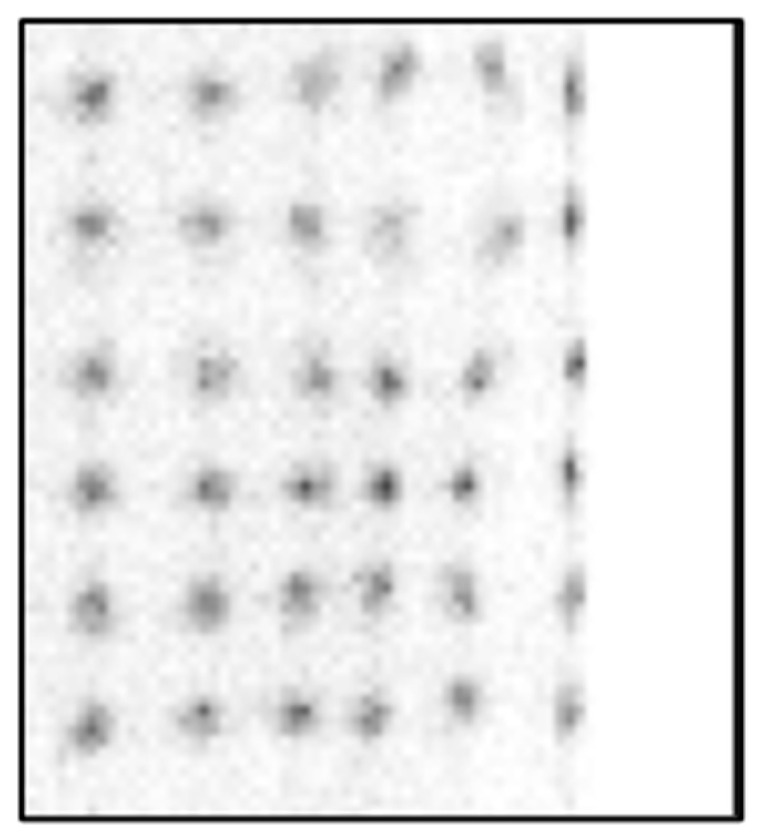
Close-up of 6 rightmost pixels for the centermost rows for crystal map created from module with partly segmented light guide with 2.5 mm slits using single channel readout and TCoG. An “f” value of 0.0605 is used.

**Figure 12:**
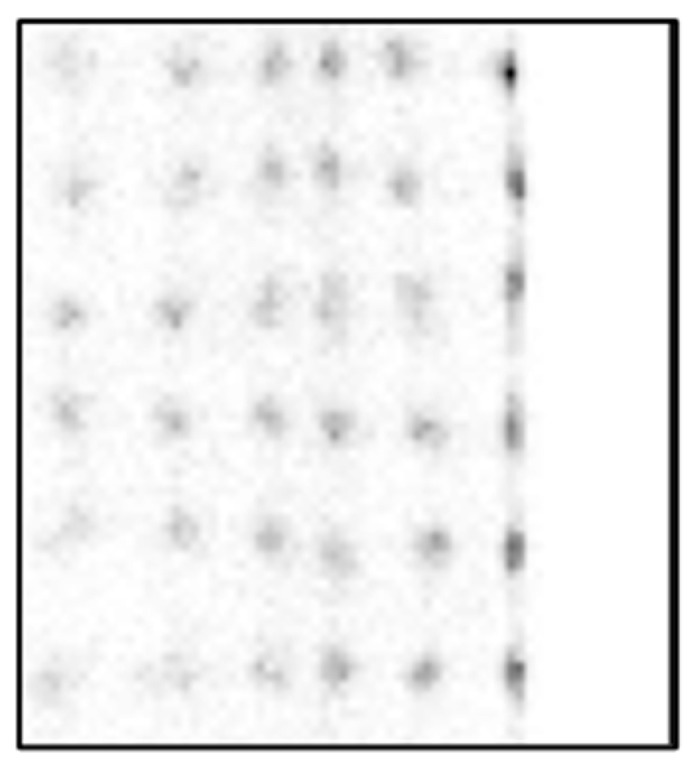
Close-up of 6 rightmost pixels for the centermost rows for crystal map created generated from module with partly segmented light guide with 3.0 mm slits using single channel readout and TCoG. An “f” value of 0.0605 is used.

**Figure 13:**
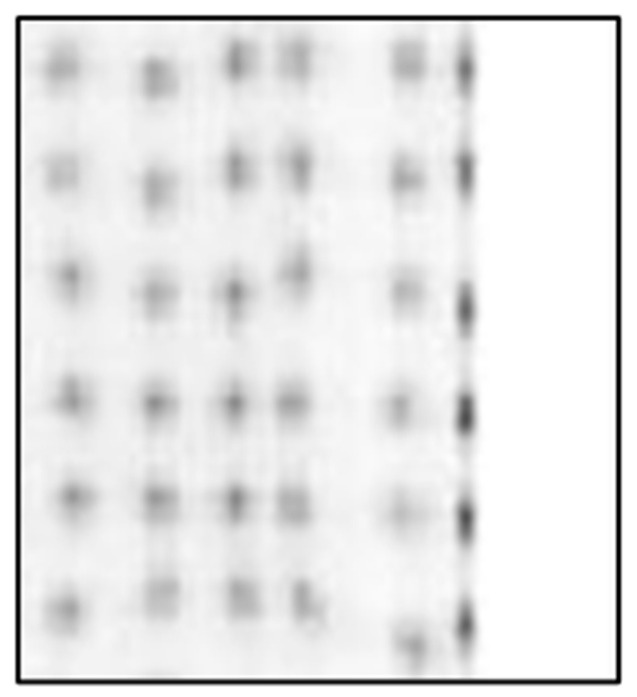
Close-up of 6 rightmost pixels for the centermost rows for crystal map created generated from module with partly segmented light guide with 4.0 mm slits using single channel readout and TCoG. An “f” value of 0.0605 is used.

Figure 11, Figure 12 and Figure 13 show zoomed images of the center of the rightmost edge of the crystal map comparing various PSLGs with different slit depths (2.5 mm, 3.0 mm and 4.0 mm, respectively) created with single channel readout and TCoG. Figure 13 reinforces that the best crystal map quality in terms of resolvability of edge pixels and representation of a true size of a crystal array is achieved with a combination of a segmented light guide with slit depth of 4.0 mm, single channel readout and TCoG.

**Figure 14:**
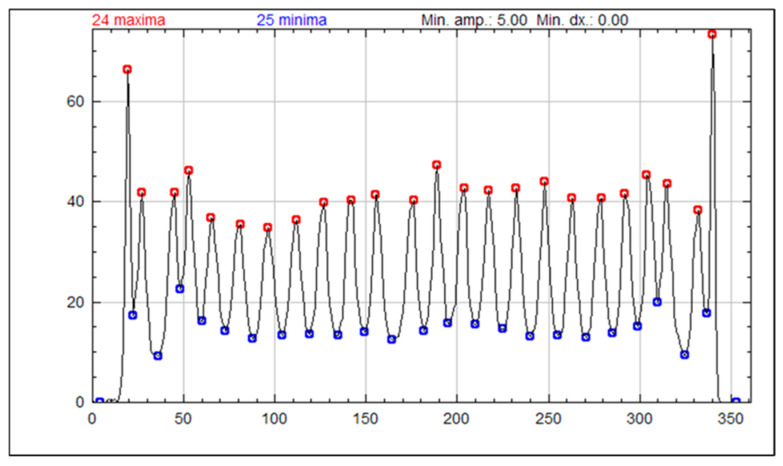
Peak profile of the averaged values of the 20 centermost rows of the crystal map generated using a partly segmented light guide with 4.0 mm slits and single channel readout and TCoG. An “f” value of 0.0605 is used.

Figure 14 contains a peak profile created from the crystal map generated using 4.0 mm PSLGs acquired with single channel readout and TCoG. For this analysis, the peak profiles were derived by averaging the values from the 20 centermost rows of the aforementioned crystal map. The resulting profile demonstrates that TCoG yields a more accurate representation of the true crystal map size, with well-separated peaks.

## 4. Discussion

The results shown in this paper focus on three main acquisition parameters: the design of the light guide used in PET sensor modules, the algorithm used in coordinate reconstruction and the signal readout method. Figure 6, Figure 7, Figure 8, Figure 9 and Figure 10 show a selection of the highest quality crystal maps generated by experimenting with different sets of parameters. The crystal map in Figure 6 is the “base case”, with a solid light guide, traditional CoG and multiplexed readout. The crystal map is significantly compressed in size compared to actual detection area, only shows 22 pixels in any given column or row and best illustrates the “edge effect” in modular PET system design due to asymmetric light distribution. TCoG algorithm (Figure 10), even with an individual channel readout, when applied to a solid light guide, provides only limited improvement in crystal map quality: pixels are more uniformly distributed and crystal map appears less compressed; however, edge pixels are not resolved.

Introducing the PSLG improves edge pixel resolvability. The ability to clearly distinguish all 24 × 24 LYSO crystal elements, regardless of whether a multiplexed or single channel readout is employed (Figure 7, Figure 8 and Figure 9), confirms the efficacy of this approach. The best results in terms of image quality and reduced noise are achieved with 4.0 mm slits filled with barium sulfate, the TCoG and single channel readout (Figure 9). It should be mentioned that traditional CoG when applied with the same PSLG and single channel readout (Figure 7) also resolves all pixels in each column and row; however, the crystal map is less uniform and slightly compressed, especially at the edges.

Experiments with different slit depths demonstrate that, when the single channel readout is combined with the TCoG for coordinate reconstruction, light guides with any of the tested slit depths (2.5 mm, 3.0 mm and 4.0 mm) allow for the edge-most pixels to be resolved (Figure 11, Figure 12 and Figure 13). The best edge pixel separation is achieved with the 4.0 mm deep slits: with this setup, the location on the crystal map most accurately represents the physical location of the LYSO crystals.

The improved edge pixel resolvability and accuracy of coordinate reconstruction using a 4.0 mm light guide, TCoG, and single channel readout is further confirmed by the peak profile for the crystal map shown in Figure 14. The profile reveals 24 clearly distinguished peaks, including the outermost pixels. While the center pixels are mostly uniform in amplitude and width, a spike in the intensity value of the edge-most pixel is apparent from the profile.

## 5. Conclusions

This study investigated the effectiveness of a PSLG design, in conjunction with various coordinate reconstruction algorithms and signal readouts, to address the persistent “edge effect” in modular SiPM-based PET detectors. Our findings demonstrate that using partially segmented light guides effectively mitigates edge effects caused by coordinate reconstruction algorithms and significantly improves crystal resolvability at the detector periphery and overall flood map quality.

We found that using slit depths between 50% and 80% of the light guide’s total thickness made all scintillation crystal pixels fully resolvable. Utilizing TCoG in conjunction with partially segmented light guides enhances the clarity of pixel signatures near sensor module edges in single channel readout systems. Increasing slit depth beyond 60% provides minimal further improvement. However, all pixel signatures remain distinctly resolved across all tested partially segmented designs. Although deeper slits create a noticeable visual gap between edge pixels and the rest of the map, this effect is purely visual and easily corrected through software-based pixel location adjustments.

Unlike other methods, segmentation of the light guide does not significantly increase manufacturing complexity or system costs. The slits can be introduced within an existing light guide, making implementation straightforward without requiring a redesign of electronic hardware. When correctly positioned, these slits effectively constrain the spread of optical photons near the detector edge, thereby improving coordinate reconstruction accuracy and reducing errors associated with center-weighted event distributions.

While the effectiveness of the PSLG is shown here for the Radialis LD-PET, the demonstrated improvements in crystal resolvability and flood map quality have broad implications beyond this specific system. Indeed, similar design can be applied to most modular, light-sharing detectors that rely on CoG or modified CoG algorithms such as TCoG or PW-CoG. Because the introduction of internal slits within the lightguide does not require changes to the detector hardware or electronics, it represents a straightforward and cost-effective upgrade for existing systems that suffer from the “edge effect”. Of course, system-specific optimizations to refine photon distribution, spatial resolution and improvement in overall detector performance will be required.

## Data Availability

Data are available from corresponding author upon reasonable request.

## Abbreviations

The following abbreviations are used in this manuscript:

PET	Positron Emission Tomography
LYSO	Lutetium Yttrium Orthosilicate
SiPM	Silicon PhotoMultiplier
TOF	Time-of-Flight
CoG	Center of Gravity
TCoG	Truncated Center of Gravity
PSLG	Partly Segmented Light Guide
LD	Low Dose
